# Omicron Variant Escapes Therapeutic Monoclonal Antibodies (mAbs) Including Recently Released Evusheld^®^, Contrary to 8 Prior Main Variant of Concern (VOC)

**DOI:** 10.1093/cid/ciac143

**Published:** 2022-02-16

**Authors:** Céline Boschi, Philippe Colson, Audrey Bancod, Valérie Moal, Bernard La Scola

**Affiliations:** Microbes, Evolution, Phylogeny et Infection (MEPHI), Aix Marseille Université, Marseille, France; Institut Hospitalo-Universitaire Méditerranée-Infection, Marseille, France; Microbes, Evolution, Phylogeny et Infection (MEPHI), Aix Marseille Université, Marseille, France; Institut Hospitalo-Universitaire Méditerranée-Infection, Marseille, France; Microbes, Evolution, Phylogeny et Infection (MEPHI), Aix Marseille Université, Marseille, France; Institut Hospitalo-Universitaire Méditerranée-Infection, Marseille, France; Microbes, Evolution, Phylogeny et Infection (MEPHI), Aix Marseille Université, Marseille, France; Department of Nephrology, Conception hospital, AP-HM, Marseille, France; Microbes, Evolution, Phylogeny et Infection (MEPHI), Aix Marseille Université, Marseille, France; Institut Hospitalo-Universitaire Méditerranée-Infection, Marseille, France

To the Editor—In their recent article, Stein et al reported efficiency of REGN-CoV-2^®^ in patients with long-standing coronavirus disease 2019 (COVID-19), allowing rapid viral clearance and clinical improvement [1]. This therapy, using monoclonal antibodies (mAbs) targeting the receptor-binding domain of the severe acute respiratory syndrome coronavirus 2 (SARS-CoV-2) spike protein, is also currently used for active immunization of COVID-19 in immunocompromised patients that do not respond to a complete vaccine schedule. We tested herein, as previously described in our institute (Supplementary Data) [2], the neutralizing activity of 6 mAbs that are authorized for clinical use, including bamlanivimab and etesevimab (alone or in combination), casirivimab and imdevimab (alone or in combination as REGN-CoV-2^®^) and tixagevimab and cilgavimab (alone or in combination as Evusheld^®^) against SARS-CoV-2 strains isolated during the pandemic. Strains are the French original B.1.1 virus and 9 variants of concern or of interest: B.1.160, Alpha (B.1.1.7), Beta (B.1.351.2), Delta original (AY.71) and of sublineage (AY.4.2), Iota (B.1.526), Epsilon (B.1.429), Mu (B.1.621), and the recent Omicron (B.1.1.529) [3]. As Evusheld received only access approval last month, as supposed to retain activity against omicron variant, we tested in only against the latest variants of concern Delta and Omicron.

We found an absence of inhibition by bamlanivimab of the Beta and Delta variants as previously reported [4] but also of Epsilon and Mu variants (Figure 1A). For etesevimab, 50% of neutralization was obtained at 0.2 µg/mL for Delta and at 0.1 µg/mL for AY4.2 variants (Figure 1A and Supplementary Data 1). We observed no neutralization by casirivimab of Beta and Mu variants (Figure 1B and Supplementary Data 1). Imdevimab neutralized all variants except Omicron but concentrations to obtain 50% of neutralization were higher on average than with casirivimab (Figure 1B and Supplementary Data 1). Unexpectedly, the casirivimab/imdevimab cocktail showed an important synergistic effect, particularly on Delta, AY4.2, and Epsilon variants because 50% of neutralization was observed at 0.03 µg/mL (Figure 1B and Supplementary Data 1). However, none of the 4 mAbs either alone or in combination neutralized the new Omicron variant (Figure 1A, 1B and Supplementary Data 1). As for the new mAbs composing Evusheld^®^, we observed 50% of neutralization by cilgavimab at 5.8 µg/mL and at 0.07 µg/mL for tixagevimab on Delta variant and at 0.03 µg/mL for the combination (Figure 1C and Supplementary Data 2). For the Omicron variant, concentration to obtain 50% of neutralization by cilgavimab were higher (1200 µg/mL) and no neutralization were obtained with tixagevimab. In combination, concentration to obtain 50% of neutralization was at 6.4 µg/mL. (Figure 1C and Supplementary Data 2). Thus, 4 of the 6 mAbs used alone or in combination in vitro showed a complete loss of their neutralizing activity against Omicron variant, a recently reported feature compared to the WA1/2020 D614G parental isolate [5]. As for Evusheld^®^, we observed a partial neutralizing activity against Omicron, and this combination was 233 times less active on Omicron than on Delta variant, suggesting limited efficiency and need for reinforcement of protective measures against infection for immunocompromised patients rather than protection with currently available mAbs.

**Figure 1.**
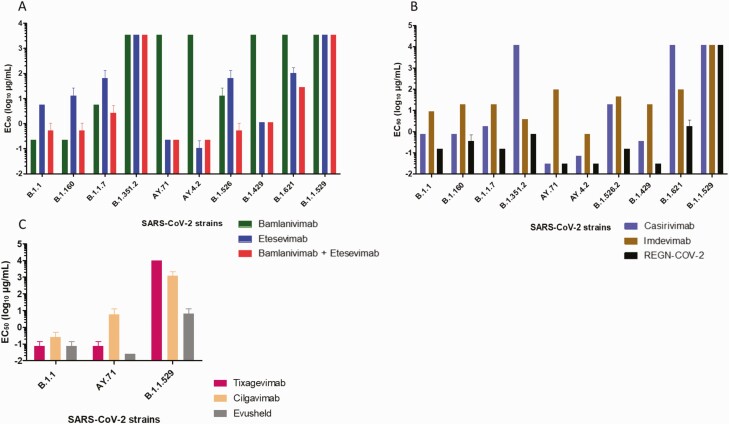
Concentrations required to obtain 50% neutralization (EC_50_ log_10_ µg/mL) for each mAb. *A*, bamlanivimab, etesevimab, mixture of bamlanivimab and etesevimab, *B*, casirivimab, imdevimab and REGN-CoV-2^**®**^ on the 10 SARS-CoV-2 strains tested. *C*, tixagevimab and cilgavimab and Evusheld^**®**^ on B.1.1 virus, AY.71, and B.1.1.529 strains. Each mAb was tested 3 times (except for B.1.1.529 variant 4 times). Bars represented the standard error. Abbreviations: mAB, monoclonal antibodies; SARS-CoV-2, severe acute respiratory syndrome coronavirus 2.

## Supplementary Data

Supplementary materials are available at *Clinical Infectious Diseases* online. Consisting of data provided by the authors to benefit the reader, the posted materials are not copyedited and are the sole responsibility of the authors, so questions or comments should be addressed to the corresponding author.

ciac143_suppl_Supplementary_Figure_S1

ciac143_suppl_Supplementary_Figure_S2

ciac143_suppl_Supplementary_Table_S3

ciac143_suppl_Supplementary_Data

## Notes

***Acknowledgments.*** The authors are indebted to Marie-Charlotte Mati and Clio Grimaldier for strains isolation, Gwilherm Penant and Priscilla Jardot for reverse transcription polymerase chain reaction (RT-PCR) and genome sequencing of isolates.

***Financial support.*** This research was funded by the French Government under the “Investissements d’avenir” (Investments for the Future) program managed by the Agence Nationale de la Recherche (ANR, French National Agency for Research) (reference: Méditerranée Infection 10-IAHU-03).

***Author contributions.*** C. B. performed microneutralization tests and cowrote the first draft of the paper, P. C. performed genomic analysis data and cowrote the first draft of the paper, A. B. performed microneutralization tests, V. M. codesigned the study, and B. L. S. conceived and codesigned the study and cowrote the first draft of the paper. All authors contributed to discussion and interpretation of the results, and to the writing of the paper. All authors have read and approved the final paper.

***Declaration of interest.*** This research was funded by the French Government under the “Investissements d’avenir” (Investments for the Future) program managed by the Agence Nationale de la Recherche (ANR, French National Agency for Research) (reference: Méditerranée Infection 10-IAHU-03).

***Potential conflicts of interest.*** B. L. S. reports scientific consultant fees from Amoéba biotech and GIS Edem solution; patents PCT/FR2015/050980 for method and liquid medium for transporting and preserving bacteria and PCT/2015/052840 for method for identifying a microbe in a clinical sample and bank of mass spectra produced by MALDI-TOF type mass spectrometry; and minor shareholder from Culture top society. All other authors report no potential conflicts. All authors have submitted the ICMJE Form for Disclosure of Potential Conflicts of Interest. Conflicts that the editors consider relevant to the content of the manuscript have been disclosed.
