# Supplementary figures and images for: Omicron Variant Escapes Therapeutic Monoclonal Antibodies (mAbs) Including Recently Released Evusheld®, Contrary to 8 Prior Main Variant of Concern (VOC)
Source: Clin Infect Dis. 2022 Feb 16;75(1):e534–5. doi: 10.1093/cid/ciac143 (PMC9402686; doi:10.1093/cid/ciac143)

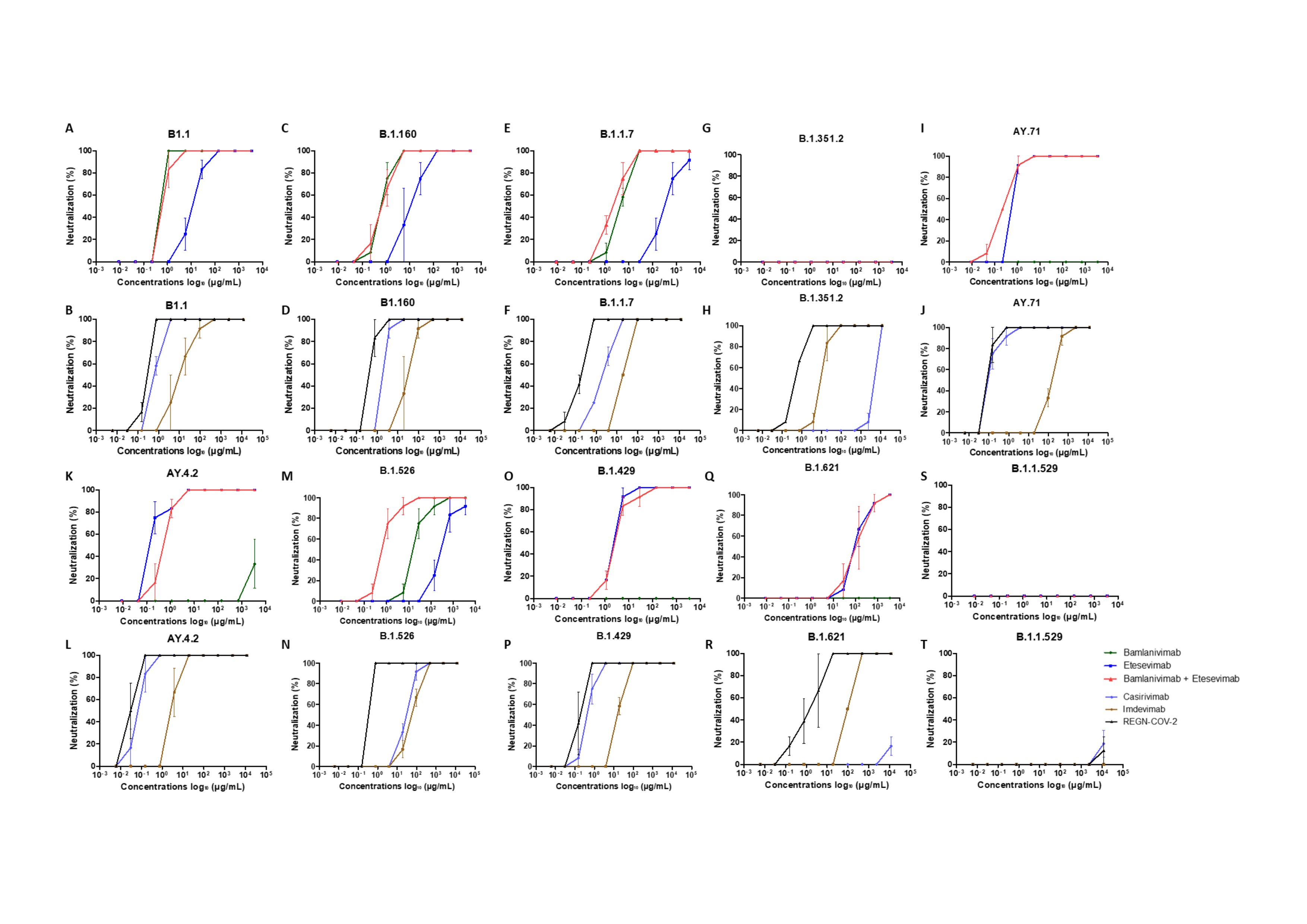

Supplement: ciac143_suppl_Supplementary_Figure_S1 [file ciac143_suppl_supplementary_figure_s1.jpeg]

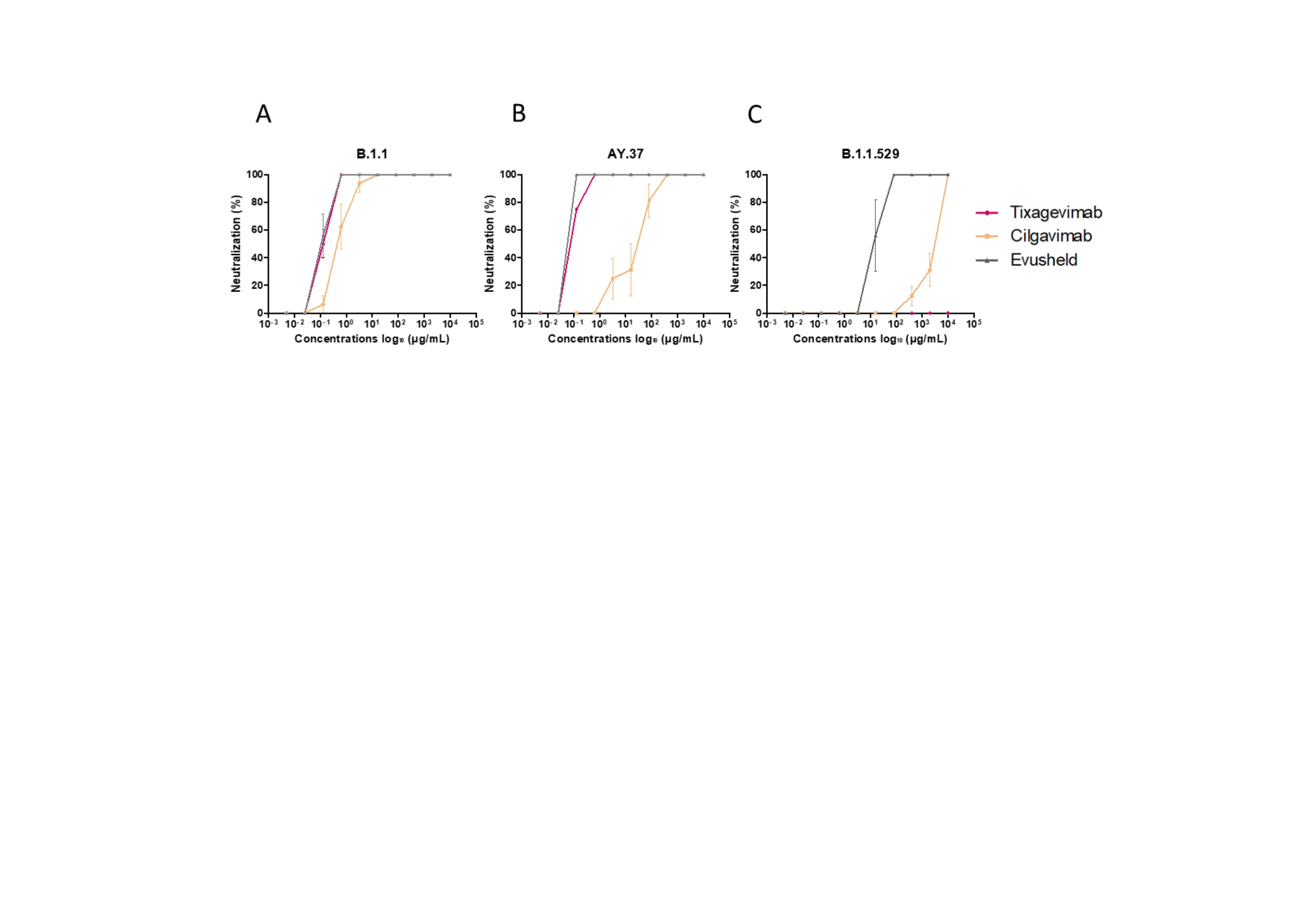

Supplement: ciac143_suppl_Supplementary_Figure_S2 [file ciac143_suppl_supplementary_figure_s2.jpeg]
