# Supplementary material for: Omicron Variant Escapes Therapeutic Monoclonal Antibodies (mAbs) Including Recently Released Evusheld®, Contrary to 8 Prior Main Variant of Concern (VOC)
Source: Clin Infect Dis. 2022 Feb 16;75(1):e534–5. doi: 10.1093/cid/ciac143 (PMC9402686; doi:10.1093/cid/ciac143)
Supplement: ciac143_suppl_Supplementary_Table_S3 [file ciac143_suppl_supplementary_table_s3.pdf]

Supplementary data S3

Sequence name (including GISAID)	Nexstrain clade	Pangolin lineage	IHU name	Nucleotide substitutions	Nucleotide deletions	Amino acid substitutions	Amino acid deletions	Nucleotide substitutions	Nucleotide deletions	Amino acid substitutions	Amino acid deletions
IHUMI-717_EPI_ISL_8033347|2020-03-26	20B	B.1.1	IHUMI-717	12	0	7	0	C241T, C313T, C3037T, A7903G, C14408T, G19518T, A23403G, G26143T, G28845T, G28881A, G28882A, G28883C		N:R191L, N:R203K, N:G204R, ORF1b:P314L, ORF1b:L2017F, ORF3a:G251C, S:D614G	
IHUMI-2096_EPI_ISL_8033348|2020-08-06	20A	B.1.160	IHUMI-2096	20	15	11	5	C241T, C3037T, C4543T, G5629T, G9526T, C11497T, G13993T, C14408T, G15766T, A16889G, G17019T, C18877T, G22992A, A23403G, G25563T, C25710T, C26735T, T26876C, G28975C, G29399A	23585-23599	N:M234I, N:A376T, ORF1a:M3087I, ORF1b:A176S, ORF1b:P314L, ORF1b:V767L, ORF1b:K1141R, ORF1b:E1184D, ORF3a:Q57H, S:S477N, S:D614G	S:Q675-, S:T676-, S:Q677-, S:T678-, S:N679-
IHUMI-3076_EPI_ISL_982232|2021-01-08	20I (Alpha, V1)	B.1.1.7	IHUMI-3076	31	25	20	8	C241T, C913T, A1399G, C3037T, C3267T, C5388A, C5986T, T6954C, C14408T, C14676T, C15279T, T16176C, A17615G, A23063T, C23271A, A23403G, C23604A, C23709T, T24506G, G24914C, G26730C, C27972T, G28048T, A28111G, G28280C, A28281T, T28282A, G28881A, G28882A, G28883C, C28977T	11288-11296, 12041-12046, 21765-21770, 21992-21994, 28271	M:V70L, N:D3L, N:R203K, N:G204R, N:S235F, ORF1a:T1001I, ORF1a:A1708D, ORF1a:I2230T, ORF1b:P314L, ORF1b:K1383R, ORF8:Q27*, ORF8:R52I, ORF8:Y73C, S:N501Y, S:A570D, S:D614G, S:P681H, S:T716I, S:S982A, S:D1118H	ORF1a:S3675-, ORF1a:G3676-, ORF1a:F3677-, ORF1a:D3926-, ORF1a:I3927-, S:H69-, S:V70-, S:Y144-
IHUMI-3147_EPI_ISL_8033349|2021-01-22	20H (Beta, V2)	B.1.351.2	IHUMI-3147	26	18	18	6	G174T, C241T, C1059T, A2692T, C3037T, G5230T, A8052G, A10323G, G10396T, C10632T, C14408T, C21614T, A21801C, A22206G, G22813T, G23012A, A23063T, A23403G, T23560C, C23664T, G25563T, C25904T, C26456T, C28253T, T28729C, C28887T	11288-11296, 22283-22291	E:P71L, N:T205I, ORF1a:T265I, ORF1a:K1655N, ORF1a:N2596S, ORF1a:K3353R, ORF1a:A3456V, ORF1b:P314L, ORF3a:Q57H, ORF3a:S171L, S:L18F, S:D80A, S:D215G, S:K417N, S:E484K, S:N501Y, S:D614G, S:A701V	ORF1a:S3675-, ORF1a:G3676-, ORF1a:F3677-, S:L241-, S:L242-, S:A243-
IHUMI-3630_EPI_ISL_8033350|2021-06-07	21I (Delta)	AY.71	IHUMI-3630	33	13	27	4	G210T, C241T, C2509T, C3037T, C5184T, A5584G, C9891T, T11418C, C11514T, C13019T, C14408T, G15451A, C16466T, C16726T, A18931G, C19118T, C21618G, G21987A, C22227T, T22917G, C22995A, A23403G, G24410A, C25317T, C25469T, C26753T, T26767C, T27638C, C27752T, A28461G, G28881T, G29402T, G29742T	22029-22034, 28248-28253, 28271	M:I82T, N:D63G, N:R203M, N:D377Y, ORF1a:P1640L, ORF1a:A3209V, ORF1a:V3718A, ORF1a:T3750I, ORF1b:P314L, ORF1b:G662S, ORF1b:P1000L, ORF1b:H1087Y, ORF1b:I1822V, ORF1b:A1884V, ORF3a:S26L, ORF7a:V82A, ORF7a:T120I, ORF9b:T60A, S:T19R, S:G142D, S:R158G, S:A222V, S:L452R, S:T478K, S:D614G, S:D950N, S:S1252F	ORF8:D119-, ORF8:F120-, S:E156-, S:F157-

Sequence name (including GISAID)	Nextstrain clade	Pangolin lineage	IHU name	Nucleotide substitutions	Nucleotide deletions	Amino acid substitutions	Amino acid deletions	Nucleotide substitutions	Nucleotide deletions	Amino acid substitutions	Amino acid deletions
IHUMI-5002_EPI_ISL_8033351|2021-10-25	21J (Delta)	AY.4.2	IHUMI-5002	40	13		4	G210T, C241T, T1391C, C1973T, C3037T, G4181T, C6402T, C7124T, C7851T, C8986T, G9053T, C10029T, A11201G, A11332G, C14408T, G15451A, C16466T, T17040C, C19220T, C21618G, C21846T, G21987A, T21995C, C22227T, T22917G, C22995A, A23403G, C23604G, G24410A, C25469T, C25614T, T26767C, T27638C, C27752T, C27874T, A28461G, G28881T, G28916T, G29402T, G29742T	22029-22034, 28248-28253, 28271	M:I82T, N:D63G, N:R203M, N:G215C, N:D377Y, ORF1a:S376P, ORF1a:A1306S, ORF1a:P2046L, ORF1a:P2287S, ORF1a:A2529V, ORF1a:V2930L, ORF1a:T3255I, ORF1a:T3646A, ORF1b:P314L, ORF1b:G662S, ORF1b:P1000L, ORF1b:A1918V, ORF3a:S26L, ORF7a:V82A, ORF7a:T120I, ORF7b:T40I, ORF9b:T60A, S:T19R, S:T95I, S:G142D, S:Y145H, S:R158G, S:A222V, S:L452R, S:T478K, S:D614G, S:P681R, S:D950N	ORF8:D119-, ORF8:F120-, S:E156-, S:F157-
IHUMI-3795_EPI_ISL_8033481|2021-07-12	21F (Iota)	B.1.526	IHUMI-3795	37	20		3	C241T, C1059T, C3037T, G6101A, A7201G, C8809T, T9867C, C14408T, A16500C, A20262G, C21575T, C21846T, A22320T, C22498T, G22992A, A23403G, A24432G, C25517T, G25563T, A25968G, C27739T, C27925T, C28311T, T28879G, G29254T, C29738A, T29753A, G29755A, G29757T, T29758G, G29759C, C29762G, A29763G, T29765A, A29768G, G29779A, T29785C	11288-11296, 28271, 29740-29745, 29749-29750, 29770-29771	N:P13L, N:S202R, ORF1a:T265I, ORF1a:G1946S, ORF1a:L3201P, ORF1b:P314L, ORF1b:Q1011H, ORF3a:P42L, ORF3a:Q57H, ORF7a:L116F, ORF8:T11I, ORF9b:P10S, S:L5F, S:T95I, S:D253V, S:S477N, S:D614G, S:Q957R	ORF1a:S3675-, ORF1a:G3676-, ORF1a:F3677-
IHUMI-4219_EPI_ISL_8033611|2021-07-22	21C (Epsilon)	B.1.429	IHUMII-4219	28	3		1	C241T, C1059T, C2395T, T2597C, C3037T, C7056A, T7057A, G7058A, G7059A, C8947T, G10282A, G11083T, C12100T, A12878G, C14408T, G17014T, G21600T, G22018T, T22917G, A23403G, T24349C, G25563T, C26681T, G27890T, G28001T, A28272T, C28887T, C29362T	518-520	N:T205I, ORF1a:T265I, ORF1a:T2264K, ORF1a:G2265N, ORF1a:L3606F, ORF1a:I4205V, ORF1b:P314L, ORF1b:D1183Y, ORF3a:Q57H, S:S13I, S:W152C, S:L452R, S:D614G	ORF1a:M85-
IHUMI-3964_EPI_ISL_8033762|2021-07-27	21H (Mu)	B.1.621	IHUMI-3964	32	4		1	C241T, C3037T, A3428G, C4878T, C5192T, C6037T, C10029T, C11344T, A11451G, A13057T, C14408T, C17491T, C17707T, C18877T, T19035C, C20148T, C21846T, A21993C, T21995A, G22599A, G23012A, A23063T, A23403G, C23604A, G24410A, G25563T, A26492T, G27008C, C27925A, C28005T, A28272T, C28887T	26158-26161	M:K162N, N:T205I, ORF1a:T1055A, ORF1a:T1538I, ORF1a:T3255I, ORF1a:Q3729R, ORF1b:P314L, ORF1b:P1342S, ORF1b:P1414S, ORF3a:Q57H, ORF8:T11K, ORF8:P38S, S:T95I, S:Y144S, S:Y145N, S:R346K, S:E484K, S:N501Y, S:D614G, S:P681H, S:D950N	ORF3a:V256-
IHUMI-5227_EPI_ISL_8033859|2021-12-01	21K (Omicron)	B.1.1.529	IHUMI-5227	54	36		15	C241T, A2832G, C3037T, T5386G, G5924A, G8393A, C10029T, C10449A, A11537G, T13195C, C14408T, C15240T, A18163G, C21762T, C21846T, G22578A, T22673C, C22674T, T22679C, C22686T, G22813T, G22898A, G22992A, C22995A, A23040G, G23048A, A23055G, A23063T, T23075C, C23202A, A23403G, C23525T, T23599G, C23604A, C23664T, C23854A, G23948T, C24130A, A24424T, T24469A, C24503T, C25000T, C25584T, C26270T, A26530G, C26577G, G26709A, A27259C, C27807T, A28271T, C28311T, G28881A, G28882A, G28883C	6513-6515, 11285-11293, 21765-21770, 21987-21995, 28362-28370	E:T9I, M:D3G, M:Q19E, M:A63T, N:P13L, N:R203K, N:G204R, ORF1a:K856R, ORF1a:V1887I, ORF1a:L2084I, ORF1a:A2710T, ORF1a:T3255I, ORF1a:P3395H, ORF1a:I3758V, ORF1b:P314L, ORF1b:I1566V, ORF9b:P10S, S:A67V, S:T95I, S:Y145D, S:G339D, S:S371L, S:S373P, S:S375F, S:K417N, S:G446S, S:S477N, S:T478K, S:Q493R, S:G496S, S:Q498R, S:N501Y, S:Y505H, S:T547K, S:D614G, S:H655Y, S:N679K, S:P681H, S:A701V, S:N764K, S:D796Y, S:N856K, S:Q954H, S:N969K, S:L981F	N:E31-, N:R32-, N:S33-, ORF1a:S2083-, ORF1a:L3674-, ORF1a:S3675-, ORF1a:G3676-, ORF9b:E27-, ORF9b:N28-, ORF9b:A29-, S:H69-, S:V70-, S:G142-, S:V143-, S:Y144-
