# Supplementary material for: Omicron Variant Escapes Therapeutic Monoclonal Antibodies (mAbs) Including Recently Released Evusheld®, Contrary to 8 Prior Main Variant of Concern (VOC)
Source: Clin Infect Dis. 2022 Feb 16;75(1):e534–5. doi: 10.1093/cid/ciac143 (PMC9402686; doi:10.1093/cid/ciac143)
Supplement: ciac143_suppl_Supplementary_Data [file ciac143_suppl_supplementary_data.pdf]

Materials and methods

Cell culture

Vero E6 cells (ATCC-CRL-1586) were cultured without antibiotics in minimal in medium (MEM, Gibco, USA) with 2 mM L-glutamine and 10% foetal bovine serum (FBS) at 37°C in a 5% CO_2 incubator. Vero E6 cells were then prepared at a concentration of $5x10^5$ cells/mL in ninety-six-well plates for the neutralization tests of SARS-CoV-2 in MEM growth medium with glutamine and 4% FBS (M4 media).

SARS-CoV-2 viral strains

The ten SARS-CoV-2 strains used in this study were isolated in cells culture and stored at -80°C from patients's nasopharyngal swabs tested SARS-CoV-2 positive in our institute IHU-Méditerranée Infection during the pandemic [1,2]. The supernatant of each strains was then harvested and was genotyped by whole genome next generation (NGS) as previously described[3] (Supplementary table S2). For the neutralization tests, we inoculated the viral strains in 96-well Vero E6 cells plate at a concentration of $5x10^5$ cells/mL. Forty-eight hours post-viral infection, viral suspension was harvested and quantified by real-time reverse-transcription RT-PCR and TCID50.

Monoclonal antibodies dilutions

Bamlavinimab and etesevimab were first diluted at 1:10 then each mAbs was diluted in a 1:5 serial dilutions (from 3500 µg/mL to 0.0089 µg/mL). For the combination of the two mAbs, we tested the mixture in the highest concentration for each mAbs alone with 2 times more etesevimab than bamlavinimab.

Casirivimab and imdevimab were first diluted at 1:10 then each mAbs was diluted each in a 1:5 serial dilutions (from 12 000 µg/mL to 0,00614 µg/mL). For the combination of these two

mAbs, we tested the mixture in the highest concentration for each mAbs alone in the same proportion.

Tixagevimab and cilgavimab were first diluted at 1:10 then each mAbs was diluted each in a 1:5 serial dilutions (from 10 000 µg/mL to 0,0512 µg/mL). For the combination of these two mAbs, we tested the mixture in the highest concentration for each mAbs alone in the same proportion

Micro-neutralization assay

Each dilution of mAbs was mixed volume by volume with each viral strains with standardized inoculum at 25 Ct as previously described[4]. The mixture of viral suspension and mAbs was incubated 1h at 37 ° C under 5% CO_2. Then, 100µl of medium in the 96-well plates was removed and 100µL of the mixture for each dilution was added in quadruplate on the Vero E6 cells. Five days post-viral infection, cytopathic effect was determined with the inverted optical microscope to determine the neutralization titer to obtain 50% of neutralization. Each mAbs and combination of mAbs were tested three times against the 9 SARS-CoV-2 strains, except for omicron variant that was tested four times.

References

[1] La Scola B, Le Bideau M, Andreani J, Hoang VT, Grimaldier C, Colson P, et al. Viral RNA load as determined by cell culture as a management tool for discharge of SARS-CoV-2 patients from infectious disease wards. Eur J Clin Microbiol Infect Dis Off Publ Eur Soc Clin Microbiol 2020;39:1059–61. https://doi.org/10.1007/s10096-020-03913-9.

[2] Jaafar R, Aherfi S, Wurtz N, Grimaldier C, Van Hoang T, Colson P, et al. Correlation Between 3790 Quantitative Polymerase Chain Reaction-Positives Samples and Positive Cell Cultures, Including 1941 Severe Acute Respiratory Syndrome Coronavirus 2 Isolates. Clin Infect Dis Off Publ Infect Dis Soc Am 2021;72:e921. https://doi.org/10.1093/cid/ciaa1491.

[3] Colson P, Levasseur A, Delerce J, Pinault L, Dudouet P, Devaux C, et al. Spreading of a new SARS-CoV-2 N501Y spike variant in a new lineage. Clin Microbiol Infect Off Publ

Eur Soc Clin Microbiol Infect Dis 2021;27:1352.e1-1352.e5. https://doi.org/10.1016/j.cmi.2021.05.006.

[4] Jaafar R, Boschi C, Aherfi S, Bancod A, Le Bideau M, Edouard S, et al. High Individual Heterogeneity of Neutralizing Activities against the Original Strain and Nine Different Variants of SARS-CoV-2. Viruses 2021;13:2177. https://doi.org/10.3390/v13112177.

Supplementary data S1: Neutralization curves in Vero E6 cells for each strains tested with each mAb : A, C, E, G, I, K, M, N, O, Q, S : bamlanivimab, etesevimab and mixture of bamlanivimab and etesevimab – B, D, F, H, J, L, N, P, R, T : casirivimab, imdevimab and REGN-CoV-2. Each experiment was done three times, except for Omicron variant four times.

Supplementary data S2 : Neutralization curves in Vero E6 cells for the three strains tested with new mAb : A : B.1.1 – B : AY.71 – C : B.1.1.529 with tixagevimab, cilgavimab and combination of both. Each experiment was done four times.

Supplementary table S3: Lineage of SARS-CoV-2 isolates and mutations in the spike protein. In this table are indicated for the ten SARS-CoV-2 strains: genome sequence submitted to GISAID databe (https://www.gisaid.org/), nexstrain clade, Pangolin lineage, IHU name isolate (IHUMI) and the corresponding nucleotide substitutions, nucleotide deletions, amino acid substitutions and amino acid deletion
